# Outcome Inelasticity and Outcome Variability in Behaviour-Incidence Models: An Example from an SEIR Infection on a Dynamic Network

**DOI:** 10.1155/2012/652562

**Published:** 2012-12-05

**Authors:** Bryce Morsky, Chris T. Bauch

**Affiliations:** Department of Mathematics and Statistics, Room 437 MacNaughton Building, University of Guelph, 50 Stone Road East, Guelph, ON, Canada N1G 2W1

## Abstract

Behavior-incidence models have been used to model phenomena such as free-riding vaccinating behavior, where nonvaccinators free ride on herd immunity generated by vaccinators. Here, we develop and analyze a simulation model of voluntary ring vaccination on an evolving social contact network. Individuals make vaccination decisions by examining their expected payoffs, which are influenced by the infection status of their neighbors. We find that stochasticity can make outcomes extremely variable (near critical thresholds) and thus unpredictable: some stochastic realizations result in rapid control through ring vaccination while others result in widespread transmission. We also explore the phenomenon of outcome inelasticity, wherein behavioral responses result in certain outcome measures remaining relatively unchanged. Finally, we explore examples where ineffective or risky vaccines are more widely adopted than safe, effective vaccines. This occurs when such a vaccine is unattractive to a sufficient number of contacts of an index case to cause failure of ring vaccination. As a result, the infection percolates through the entire network, causing the final epidemic size and vaccine coverage to be higher than would otherwise occur. Effects such as extreme outcome variability and outcome inelasticity have implications for vaccination policies that depend on individual choice for their success and predictability.

## 1. Introduction

With the advent of vaccines, medicines, and improved hygiene and sanitation, disease burden has decreased considerably compared to previous centuries [1]. However, the modern era has brought new problems. For example, many infectious diseases caused by bacteria are becoming resistant to antibiotics [2]. Immunization programs are likewise facing challenges: in some cases, the primary barrier to maintaining high vaccine coverage is no longer lack of access to vaccines but rather lack of population uptake of vaccination. For example, unfounded vaccine scares have set back eradication programs and caused outbreaks of disease in countries that were previously close to eliminating the infections [3].

Traditional infectious disease transmission modeling assumes homogeneous mixing of the population, that is, individuals interact with all other individuals within the population equally; there is no preferential interaction. As such, any susceptible individual can become infected directly via contact with any infectious individual in the population [4, 5]. However, heterogeneous mixing is the reality in many situations.

One way we can conceptualize this in terms of individuals being part of social contact networks [6]. A social network is composed of nodes that are attached to one another with edges. Each node in the network represents a decision maker in the population. The edges that connect nodes represent the social connections of these individuals. These connections can represent contact between family members living with one another, friends, coworkers, and schoolmates. Infection spreads from node to node along these edges. Compared to a homogeneous mixing population, this structure increases the danger of infection to any node sharing an edge with an infectious node, whereas nodes not in direct contact with an infectious node are not in danger of infection until the infection reaches them from elsewhere in the network.

The effectiveness of individuals voluntarily choosing to be vaccinated to control the spread of infectious diseases can rely upon how individuals weigh the risk of vaccinating versus the risks associated with infection, and this tension can be modeled using game theory or related behavioral modeling approaches [7–17]. We can view the choices to be vaccinated and to refrain from vaccination as strategies. And, whichever of these strategies results in less adverse health impact is the strategy we assume an individual will choose. The choices that individuals make also affect the population as a whole. If more individuals are vaccinated, herd immunity can protect those who are susceptible in the population. However, susceptible individuals can “free ride” on the herd immunity generated by vaccinated individuals, who accepted real or perceived risks to get vaccinated. This effect results in a conflict between private and group interests: a social dilemma. This effect can be seen as an example of *policy resistance*, which is the tendency for human behavioral responses to a newly introduced intervention to undermine the intervention [18]. Policy resistance is an example of a nonlinear feedback phenomenon.

Some game theoretical models assuming a homogeneously mixed population predict that an infectious disease cannot be eradicated due to this social dilemma [9, 19]. If it is assumed that there is some risk to being vaccinated, the proportion of the population that vaccinates will always be less than the proportion required for elimination of the infection due to herd immunity. Therefore, unless the risk from being vaccinated is zero, the infection cannot be contained. However, the global eradication of smallpox in 1977 despite voluntary vaccination policy in many jurisdictions contradicts this prediction [20, 21].

A previous game theoretical model of vaccinating behavior on a contact network identified one possible solution to this paradox, by showing that constraining transmission to occur on a contact network changes the incentive to vaccinate and makes rapid control of an outbreak through voluntary ring vaccination possible [22, 23]. Ring vaccination is a strategy of preventing transmission of infection by vaccinating the contacts of an infectious index case. Smallpox was spread primarily through close interactions between individuals, hence a network approach to smallpox transmission is applicable, and ring vaccination was used in the final stages of the smallpox eradication program [20, 21]. Numerous publications have shown the many other ways in which epidemic dynamics on a network, with or without behavioral modeling included, can diverge from epidemic dynamics in a homogeneously mixing population [24–30].

The network-based behavior-incidence models of [22, 23] assumed a static contact network. Here, we use a simulation model to explore vaccinating behavior during an epidemic on a dynamically changing contact network, representing the changing social connections that characterize real populations on both shorter and longer timescales. We assume a hypothetical close contact infection, such as one spread through household contacts and in healthcare settings, making use of a social contact network appropriate. We identify parameter regimes that give rise to interesting dynamics that are relevant to disease control efforts. For instance, we identify regimes where stochastic effects can lead to enormous variability in outcomes, suggesting that the outcome of a voluntary immunization program would be highly unpredictable. We also explore examples of policy resistance [18]. Finally, we illustrate the concept of outcome inelasticity, whereby the nonlinear feedbacks inherent in behavior-incidence dynamics cause certain outcome measures to remain relatively constant across a broad range of parameter values. Our objective is not to directly inform public health policy as it relates to control of infection through ring vaccination. Rather, we are interested in exploring the range of possible dynamics exhibited in simple models of this type.

## 2. Methods

### 2.1. Initial Network Construction

We construct a social network composed of *N* nodes with mean node degree *ν*. Each node is an individual. During the construction phase of the simulation, we select two nodes at random and place an edge between them if they are not already connected. We continue until *νN*/2 edges in total have been placed. It can be shown that the resulting node degree distribution is Poisson. The edges represent a social contact between the individuals in the network through which infection can spread, such as contacts between family members or between healthcare workers and patients. We will describe the edge turnover algorithm in a Section 2.4.

### 2.2. Disease Natural History

We consider a hypothetical acute, self-limiting infection, with a latent and infectious period, long-lasting natural immunity, high rate of symptomatic infections, and a high mortality rate. Infection is followed by a latent period where the infected individual is not yet infectious; the latent period is followed by an infectious period where the individual is both symptomatic and infectious. There is a probability *β* per day that a susceptible node is infected by an infected neighbor. Thus, for a node with *n*_inf⁡_ infectious neighbors, the probability *λ* of being infected per day is

(1) $$\begin{array}{c} \lambda =1-{\left(1-\beta \right)}^{n_{\inf}}. \end{array}$$

We assume that a node only becomes infectious after a latent period described by a Gamma distribution with mean 1/*σ* and variance *Ω*_*σ*_, and remains infectious for some period also determined by a Gamma distribution with mean 1/*γ* and variance *Ω*_*γ*_. We assume that the start of infectiousness coincides with the start of symptomaticity, that all infectious nodes exhibit symptoms, and that a node is aware if any of its neighbors are exhibiting symptoms.

The vaccine carries a low but non-negligible mortality risk. For simplicity, we will assume that a susceptible node who has been efficaciously vaccinated is immediately protected whereas the vaccine has no effect in nodes who are already infected. Every node who wants to be vaccinated can be vaccinated, but vaccine is only available to neighbors of an infected node. The all-or-none vaccine efficacy is *ϵ*.

### 2.3. The Vaccination Decision Process

In each timestep, every susceptible unvaccinated node has the option to be vaccinated or to refrain from being vaccinated (hence, we assume a population where outbreak investigations entailing contact tracing are possible and vaccines are available). Each node attempts to maximize its payoff. Thus, to determine whether or not a node chooses to be vaccinated, a node's payoff for vaccinating, *P*_*V*_, is compared to its payoff for not vaccinating, *P*_*N*_. If *P*_*V*_ > *P*_*N*_, then the node is vaccinated, and otherwise the node refrains from being vaccinated (although the node may be vaccinated in the future). The formulae for *P*_*N*_ and *P*_*V*_ are detailed in (2) and (3) and the payoff functions are similar to those of [22, 23].

Under some circumstances, such as prophylactic vaccination, individuals need not wait to get vaccinated until a contact has been identified as being infectious, and so our assumption that nodes only seek vaccination when a contact is infected would not apply. However, for ring vaccination, there are scenarios where this assumption would be valid. For instance, public health may restrict the option to get vaccinated to contacts of an identified index case, due to short vaccine supply or insufficient front-line staff to vaccinate everyone who wants a vaccine.

The payoff functions are expressed in units of life years, that is, the number of remaining years that the node can expect to live. Let *L* be a baseline payoff, corresponding to the expected number of life years remaining before penalties from vaccination or infection are taken into account. We first derive the payoff for not being vaccinated. If a node escapes being infected in a given day but remains susceptible and thus may be infected in future, it receives a payoff of *α*. Normally, we expect *α* < *L* since if a node remains susceptible, it is at risk of being infected in the future if it comes into contact with an infectious neighbor, and hence their payoff should be less than guaranteed perfect health *L*. However, we set *α* = *L* because (1) this is a conservative assumption with respect to testing the effectiveness of voluntary ring vaccination, since if ring vaccination is effective for *α* = *L*, it will be effective for *α* < *L*, and (2) it is difficult to quantify how much less *α* is than *L*.

If a node is infected, then their penalty is a probability *d*_inf⁡_ of dying each day that they are infected. A node who dies from infection receives a payoff of 0, and a node who survives infection and is thereby conferred with lifelong immunity receives a payoff of *L*. Therefore, since the probability of being infected per day is *λ*, the payoff for not vaccinating today is

(2) $$\begin{array}{c} P_{N}=\left(1-\lambda \right)\alpha +\lambda \left(1-d_{\inf}\right)L. \end{array}$$

This payoff function takes into account how rational individuals weigh their chance of infection according to the probability *λ*, and the penalties of either remaining susceptible and being uncertain about future prospects or the high risk of mortality associated with infection (*d*_inf⁡_).

For a node that chooses to vaccinate, we must first determine if the node dies from the small probability of life-threatening complications due to vaccination. This occurs with probability *d*_vac_. We chose a baseline value *d*_vac_ = 0.001. This value is much higher than what is realistic for existing vaccines. However, our primary objective was to explore the possible dynamics in various parts of the parameter space of the model, and more realistic values for *d*_vac_ would have resulted in only one outcome—rapid control through voluntary ring vaccination—making the analysis trivial. As for all parameters in the model, we also assume that the node's perceived risk matches the actual risk *d*_vac_, in other words, individuals have “perfect information”. We also ran simulations where perceived and actual risk could differ, but we did not find any significant qualitative differences in dynamics. If death due to the vaccine occurs, then the node accrues 0 life-years; if it does not, then a determination of the effectiveness is assessed. If the vaccine is effective and nonlethal, then the node accrues *L* life-years. The probability of this event is *ϵ*(1 − *d*_vac_). If the vaccine is neither effective nor lethal, then the possible outcomes are the same as for those who did not vaccinate. If the node avoids infection, it attains *α* life years, otherwise it becomes infected and attains *L* life-years if it survives or 0 life years if it does not. The probability of the vaccine failing and being nonlethal, and the node avoiding infection is (1 − *ϵ*)(1 − *d*_vac_)(1 − *λ*). The probability of the vaccine failing and being nonlethal, and the node surviving infection is (1 − *ϵ*)(1 − *d*_vac_)*λ*(1 − *d*_inf⁡_). Thus, after some simplifications, *P*_*V*_ is

(3) $$\begin{array}{c} P_{V}=\left(L\epsilon +\left(\alpha \left(1-\lambda \right)+L\lambda \left(1-d_{\inf}\right)\right)\left(1-\epsilon \right)\right)\left(1-d_{\text{vac}}\right). \end{array}$$

Because we are modelling a single outbreak in a population with no previous history of infection or vaccination, we assume that each node has no memory of past infections or vaccinations.

### 2.4. Model Dynamics

The following algorithm describes the sequence of possible events that are applied to the population each time step (a time step is assumed to last one day).

1. Randomly select a node that has not been selected this day.
2. The following occur in a random order.
  - If the node is susceptible and has not chosen to be vaccinated in a previous time step, payoffs are calculated and the node may choose to be vaccinated. If the node is vaccinated, it may die from vaccine complications, and any edges connected to it are removed from the network. If the node is efficaciously vaccinated, it is fully protected from infection. If the node is inefficaciously vaccinated, then it will remain susceptible, but will not be able to be vaccinated again.
  - If the node is susceptible, the node may be infected by its infectious neighbors.
  - If the node has been infected and the disease is latent, the latent period may end.
  - If the node is infectious, it may die due to the infection and any edges connected to it are removed from the network.
  - If the node is infectious, it may recover with life-time immunity to the infection.
3. Repeat steps 1 and 2 until all nodes have been selected once and only once.
4. *ζ* *νN*/2 edges are destroyed.
5. *ζ* *νN*/2 edges are created by randomly selecting nodes not already connected, and creating an edge between them.
6. End time step.

The purpose of using asynchronous updating (randomizing the order of potential events that may occur) was to prevent artificial correlations from developing. As a result of this asynchronous updating, we note for example that a node may become infected either before or after it chooses to vaccinate, depending on the ordering chosen on that timestep.

We designate *ζ* as the proportion of edges in the network that are changed per day. Thus, *ζ* *νN*/2 edges are created and *ζ* *νN*/2 edges are destroyed each day. The process begins by randomly choosing a node, and breaking its connection to one of its neighbors, chosen at random. The process continues until *ζ* *νN*/2 connections have been broken. Nodes are then chosen at random to be connected to randomly chosen nodes to which they are not currently connected until *ζ* *νN*/2 connections have been made.

As initial conditions, *I*_0_ randomly selected nodes are infected and the remainder are fully susceptible and unvaccinated. For all of our simulations, we set *I*_0_ = 10 nodes, and *N* = 5000 nodes. We ran 50 simulations for each set of parameter values we examined, averaged the results, and computed the standard deviations. Each simulation ran for 300 simulated days. We chose 300 days because all simulated outbreaks ended or nearly ended by day 300.

## 3. Results and Discussion

### 3.1. Description of Dynamical Regimes

**Figure 1:**
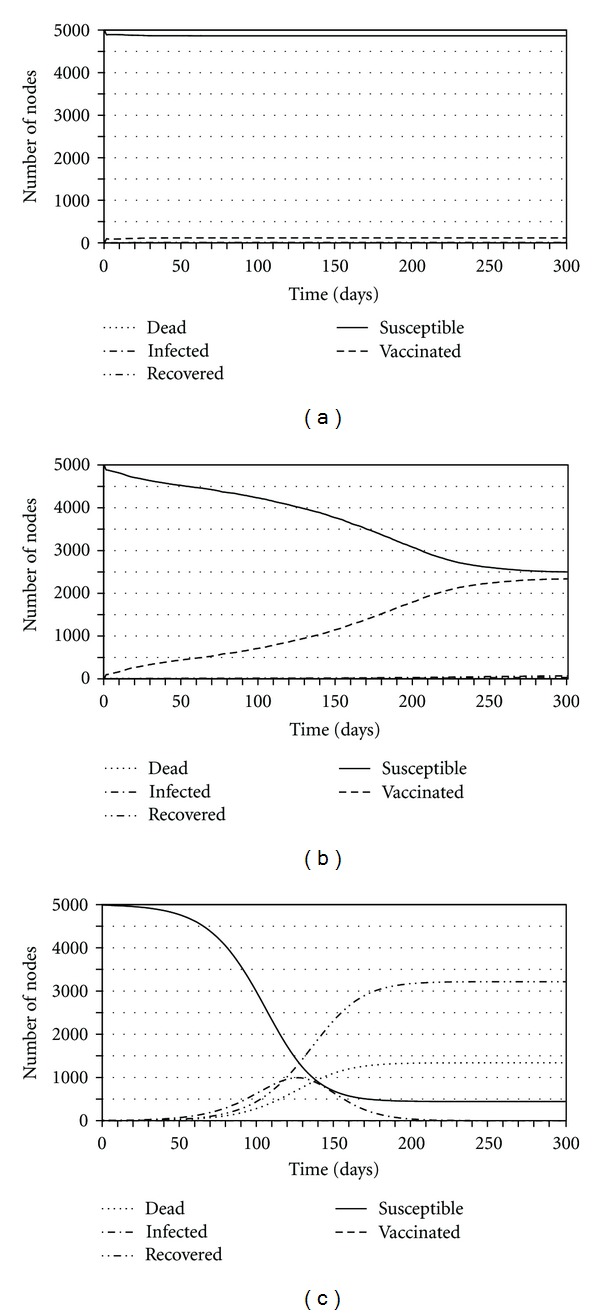
Three dynamical regimes of the model: ring vaccination (a), widespread vaccination (b), and no vaccination (c). Lines represent the number of susceptibles (solid), efficaciously vaccinated (dashed), dead (dotted), infected (fine dashed), and recovered (1 dash-2 dots) individuals. Baseline parameter values from Table 1 are used except that *d*_vac_ = 1 for the no vaccination regime. For the widespread vaccination regime, *ζ* = 0.1; for all others *ζ* = 0.

 The baseline parameter values used for all simulations appear in Table 1. Simulations use these values by default, unless otherwise noted in the figure captions. We note there are at least three dynamical regimes that can arise in this model (these are depicted in Figure 1 and summarized in Table 2).

- *Successful ring vaccination*: ring vaccination is successful and infection is quickly contained.
- *Widespread vaccination*: there is widespread vaccination and infection is either moderate or limited/contained.
- *No vaccination*: no nodes choose to get vaccinated, and there is widespread infection.

**Successful Ring Vaccination**

Ring vaccination is successful when payoff values are such that most or all neighbors of an index case immediately vaccinate, and vaccine efficacy is sufficiently high. This case occurs with the parameter values in Table 1 and when *ζ* = 0: at the parameter values of Table 1, it is optimal for neighbors to choose vaccination, and since *ζ* = 0, there is no edge turnover and hence no opportunity for the index case to come into contact with additional susceptible individuals. In this case, the infection is quickly contained. It remains possible that a few of the neighboring nodes are infected before they are vaccinated or because the vaccination was not efficacious. However, ring vaccination around these secondary cases will result in rapid containment of additional spread. This regime is also described in [23], which studies static networks.

**Widespread Vaccination**

**Figure 2:**
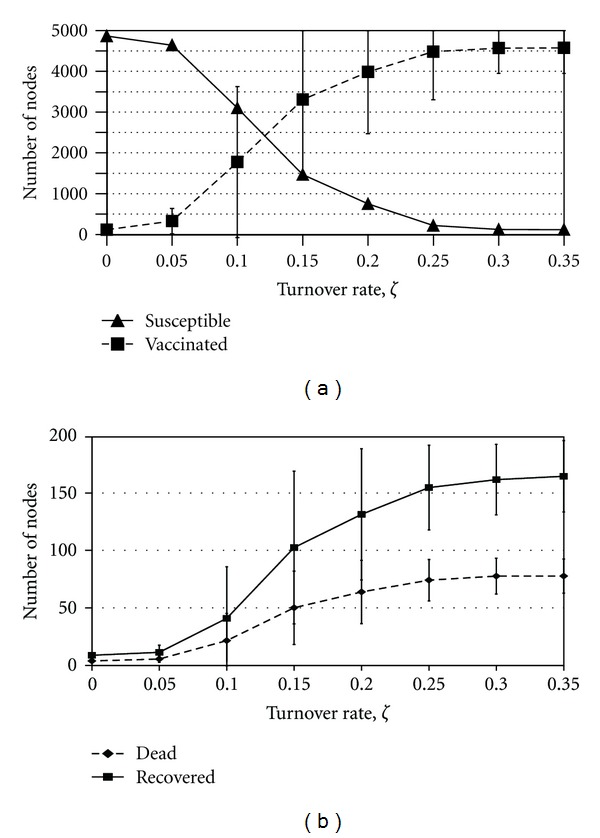
The effect of changing the turnover rate, *ζ*. Subpanel (a) shows the average final sizes of the number of susceptible (solid line) and efficaciously vaccinated (dashed line); subpanel (b) shows the average final sizes of the number of recovered (solid line) and dead (dashed line) individuals only, for the same parameter values but a different vertical scale. Bars represent one standard deviation across the 50 realizations. The standard deviation bars for susceptible nodes are not shown for clarity; they are close in magnitude to those for the number vaccinated.

**Figure 3:**
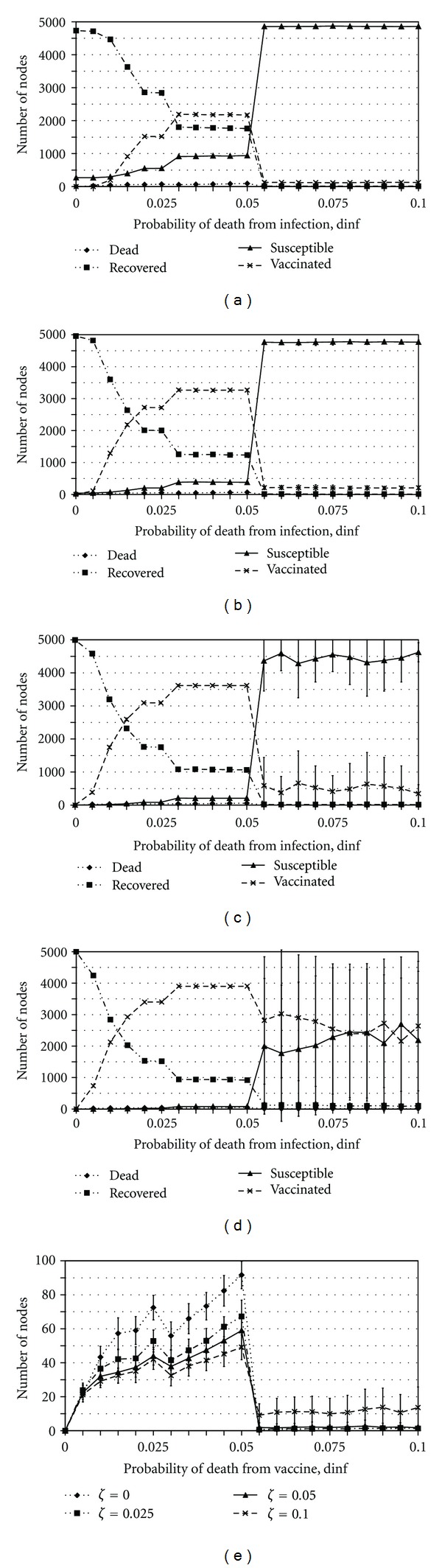
The effect of varying *d*_inf⁡_ on the average final sizes of the number of susceptible (solid line), recovered (1 dash-2 dots line), efficaciously vaccinated (dashed line), and dead (dotted line) individuals for *ζ* = 0 (a), *ζ* = 0.025 (b), *ζ* = 0.05 (c), and *ζ* = 0.1 (d), and the effect of varying *d*_inf⁡_ on the average final sizes of the number of dead individuals only, for several values of *ζ* (e). Bars represent one standard deviation across the 50 realizations.

**Figure 4:**
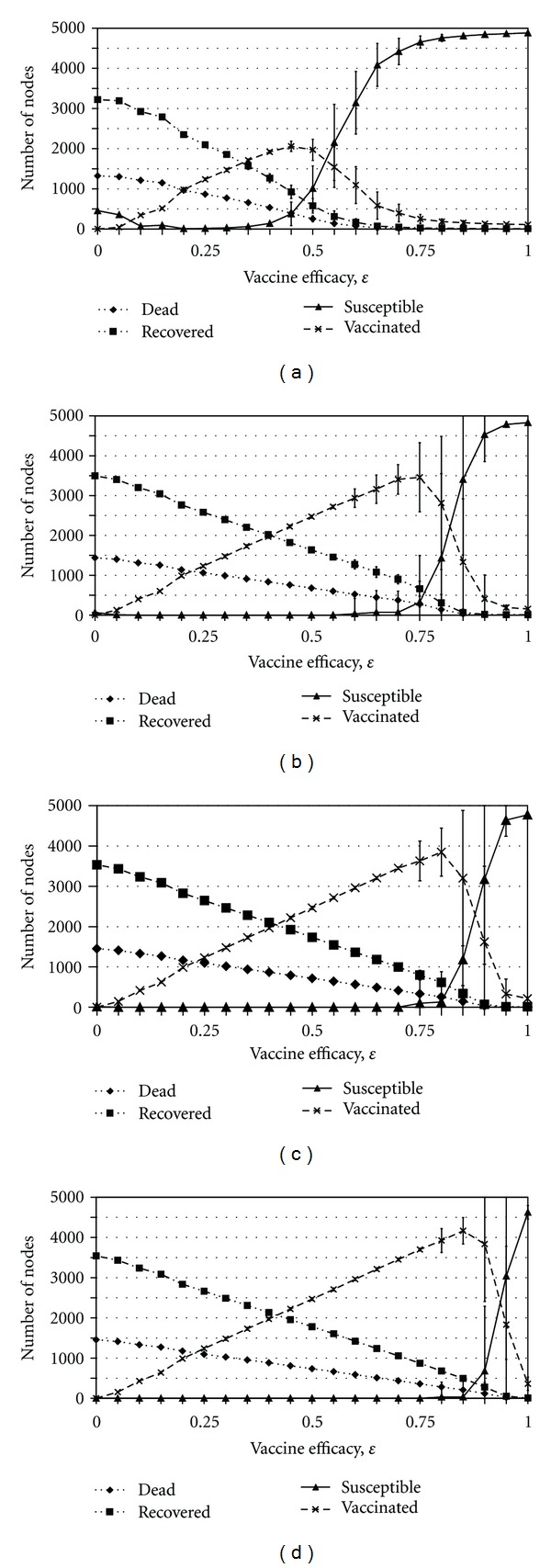
The effect of varying *ϵ* on the average final sizes of the number of susceptible (solid line), recovered (1 dash-2 dots line), efficaciously vaccinated (dashed line), and dead (dotted line) individuals for *ζ* = 0 (a), *ζ* = 0.025 (b), *ζ* = 0.05 (c), and *ζ* = 0.1 (d). Bars represent one standard deviation across the 50 realizations.

**Figure 5:**
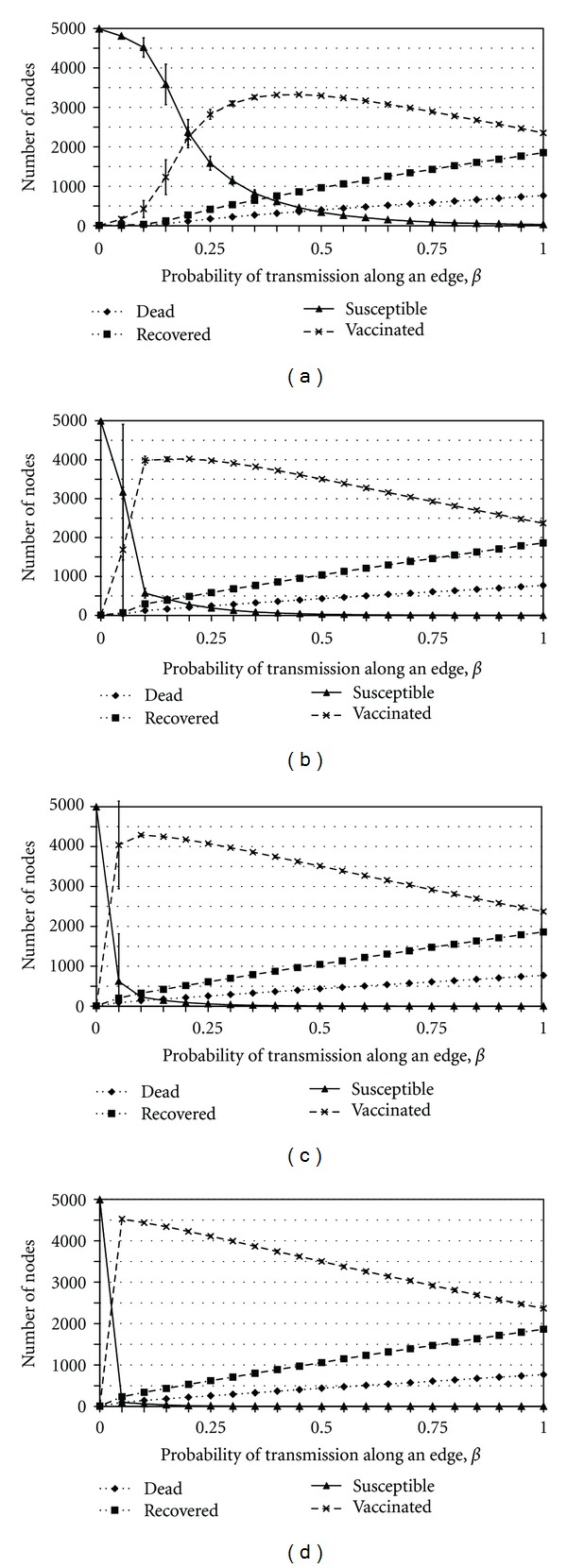
The effect of varying *β* on the average final sizes of the number of susceptible (solid line), recovered (1 dash-2 dots line), efficaciously vaccinated (dashed line), and dead (dotted line) individuals for *ζ* = 0 (a), *ζ* = 0.025 (b), *ζ* = 0.05 (c), and *ζ* = 0.1 (d). Bars represent one standard deviation across the 50 realizations.

Vaccination can become widespread when the network is sufficiently dynamic, when payoff functions are such that a node will not vaccinate unless two or more of its neighbors are infectious (implying failure of ring vaccination around a single index case), when *β* is sufficiently high, or when *ϵ* is sufficiently low. In the case where a high turnover rate is the cause of widespread vaccination (Figure 2), the outbreak spreads throughout the network as infectious nodes are continuously connected to new susceptible nodes via the network turnover effect and the susceptible nodes choose to vaccinate to protect themselves; the number vaccinated slowly rises, and the number susceptible slowly decreases until the outbreak dies out. The number of infected individuals remains limited since new neighbors of an infectious node quickly choose to vaccinate. When the cause of widespread vaccination is that two or more infectious neighbors are required to induce vaccination, infection spreads immediately beyond isolated index cases because a significant number of neighbors choose not to vaccinate, since most individuals only have zero or one infectious neighbor in the early stages of the outbreak. As a result, the infection spreads throughout the network, inducing further vaccination as well as further cases of infection. Eventually, there are enough infectious nodes to induce widespread vaccination to further halt the spread of the infection and protect the portion of the population that is susceptible. Therefore, there may still remain many susceptible nodes since vaccine is partly effective in disrupting transmission in the network. An example of a parameter range in which two infectious neighbors are required before a node will choose to be vaccinated is when 0.03 < *d*_inf⁡_ < 0.05 as can be seen in Figure 3(a). In the third case (Figure 5), where widespread vaccination is caused by the value of *β* being very high, the outbreak can effectively escape any vaccination rings and spread to many nodes simply because the disease is transmitted before neighbors have a chance to get vaccinated. Yet, the vaccination rings do prevent a degree of spread, and thus eventually, the outbreak is eventually contained. In the final case, where widespread vaccination is caused by the value of *ϵ* being very low, the infection can spread to those nodes that remain susceptible after unsuccessful vaccination. In Figure 4(a) we see a high vaccine uptake when vaccine efficacy is approximately 0.5.

**No Vaccination**

The third dynamical regime is when the payoff functions are valued such that no nodes choose to become vaccinated. When this occurs, the infection spreads throughout the network, infecting most nodes. However, due to random chance and the possibility of nodes dying before they can spread the infection to areas of the network that contain susceptible nodes, there may remain some nodes that are susceptible once the outbreak has ended.  Figure 1(c) depicts the case where the vaccine has such a high *d*_vac_ that no vaccinations occur. If we were instead to significantly reduce *d*_inf⁡_, we would observe a similar infected curve, yet would see significantly fewer deaths and more recovered.

### 3.2. Impact of Network Dynamics

Allowing network edges to turn over allows infectious individuals to escape containment rings and interact with other individuals in the population, thereby resulting in more vaccination within the population (although not necessarily more infection). For low values of the turnover rate *ζ*, we obtain the first dynamical regime where ring vaccination can be successful—the rate of turnover is not sufficient to threaten the integrity of the ring vaccination strategy. However, as we increase *ζ* and the network becomes more dynamic, more individuals come into transient contact with the index cases, causing them to vaccinate and resulting in a significant increase in the number of individuals vaccinated (Figure 2). The dependence of the final number of vaccinated and susceptible individuals on the turnover rate *ζ* is a sigmoidal curve: the number of individuals vaccinated initially grows exponentially in *ζ*, with a corresponding decrease in the number of susceptible individuals, but starts to saturate for larger values of *ζ*. The number of susceptible and vaccinated nodes are equal at roughly *ζ* = 0.12 (Figure 2). As *ζ* → 1.0, the entire population faces the decision about whether to seek vaccination. Thus, at the parameter values in Table 1, such that any individual with at least one infected neighbor chooses to vaccinate, the proportion that successfully vaccinate for high *ζ* will approximately equal *ϵ*, the proportion that die will be approximately *d*_inf⁡_(1 − *ϵ*), and the proportion that will recover will be approximately (1 − *d*_inf⁡_)(1 − *ϵ*).

### 3.3. Outcome Variability

For values of *ζ* between 0.1 and 0.2 and with other parameters are at baseline values, there is an enormous degree of variability of possible outcomes, indicated by the large standard deviations in the final sizes of the number of susceptible and vaccinated individuals (Figure 2). Within this range of outcomes occur situations where ring vaccination rapidly contains the outbreak and the final number of vaccinated individuals is therefore low, or situations where widespread vaccination occurred due to some limited secondary transmission and sufficiently rapid turnover. Such parameter regimes are relevant to public health policy, since the variability would make it difficult to predict what course an epidemic would take and whether a voluntary vaccination policy would be effective at containing the infection without the need to immunize a large proportion of the population.

The variability suggests that the system is on the boundary between the two regimes of rapid control through ring vaccination and widespread vaccination, where stochastic effects may easily tip the system in one direction or another. Hence, this may reflect proximity to an underlying critical point [30]. A significant amount of variability can also be found in other parameter regimes, including biologically plausible values for the vaccine efficacy (Figures 3, 5, and 4).

### 3.4. Outcome Inelasticity

Across a broad range of values for the turnover rate *ζ*, when the probability of death due to infection, *d*_inf⁡_, is smaller than approximately 0.05, the penalty for being infected is sufficiently small that two or more infectious neighbors are required to induce a node to be vaccinated. As a result, infection may spread widely throughout the network and many individuals may choose to be vaccinated (Figures 3(a), 3(b), 3(c), and 3(d)). The final number of recovered and vaccinated individuals is thereby large but the total number of deaths is relatively low since *d*_inf⁡_ is small. In comparison, when *d*_inf⁡_ > 0.05, the penalty for becoming infected is sufficiently large that all neighbors of an index case are induced to vaccinate and the outbreak is contained by ring vaccination (Figures 3(a)–3(d)). As a result, the final number of recovered, dead, and vaccinated individuals remain low even as the final number of susceptible individuals is high. The delineation between these two parameter regimes (widespread vaccination versus ring vaccination) is very sharp. We note that the removal of individuals for *d*_inf⁡_ > 0 can potentially influence dynamics since death due to infection will reduce the duration of infectiousness. This effect could be significant for larger values of *d*_inf⁡_ and would tend to reduce the final size.

Within the widespread vaccination regime, for *d*_inf⁡_ < 0.05, the final number of dead individuals is relatively insensitive to changes in the probability of death *d*_inf⁡_ for all values of *ζ* simulated (Figure 3(e)). In fact, as *d*_inf⁡_ increases by a factor of 10 from 0.005 to 0.05, the number of dead individuals only doubles for the higher values of *ζ*. As *d*_inf⁡_ increases, the payoff to remain unvaccinated declines and hence more individuals chose to vaccinate, which partly counteracts the effect of an increased probability of death. The nonlinear feedback loop associated with behavioral responses to disease incidence acts to maintain the number of deaths at a relatively constant level, even as the other outcomes change. We call this *outcome inelasticity*, and note that it has been observed in behavior-incidence models in other contexts [31].

### 3.5. Socially Suboptimal Outcomes and Policy Resistance

Several changes to parameters that make vaccines less attractive can increase vaccine coverage, by causing ring vaccination to fail and thus allowing infection to spread throughout the network, spurring more vaccinations in the long term.

For instance, higher risks associated with the vaccine (higher *d*_vac_) can result in more individuals choosing to be vaccinated: a higher vaccine risk results in fewer contacts of the index case choosing to vaccinate. Therefore, ring vaccination fails to contain the outbreak and the infection can spread beyond the index cases to the rest of the population. As prevalence of infection increases, more individuals have two or more infectious neighbors, and for appropriate parameter values, these individuals would now become vaccinated, when having only one infectious neighbor was insufficient to convince them to become vaccinated. Therefore, the final number of vaccinated and infected individuals is higher. A socially optimal outcome that resulted in fewer deaths within the population (and less vaccination) could have been reached had ring vaccination of the index cases been complete, but this is prevented by aversion to vaccine risks which ironically results in both higher vaccine coverage and more infected cases. This can be seen as illustrating policy resistance [18] in the sense that a policy recommendation of ring vaccination of index cases would be rendered ineffective by the individually optimal solution of avoiding vaccine risks, despite that causing the infection to spread to the rest of the population.

In a similar vein, when vaccine efficacy is very high *ϵ* ≈ 0.95, ring vaccination is successful and the outbreak is quickly contained without having to vaccinate a large proportion of the population. However, as *ϵ* decreases, more secondary transmission occurs in unsuccessfully vaccinated individuals and the final number of vaccinated and recovered individuals increases (Figure 4). Hence, a less effective vaccine is utilized more widely. However, if the vaccine efficacy decreases too much, individuals stop vaccinating altogether since the vaccine is unlikely to protect against infection and yet carries some risk (Figure 4).

### 3.6. Other Results

Some previous behavior-incidence models for SEIR-type infections that assume homogeneous mixing have found that increasing the transmissibility of the infection will increase vaccine coverage [9]. Interestingly, the opposite can occur in this model. As the edgewise transmission probability *β* is increased, a point is reached at which the number of vaccinated individuals begins decreasing as *β* continues to increase (Figure 5). At the same time, the final sizes of dead and recovered individuals continues to increase with increasing *β* (Figure 5). This effect is not due to behavioral changes, since all nodes will chose to be vaccinated if they have at least one infectious neighbor when *β* ≥ 0.0035/day. Rather, it occurs because of the close contact nature of infection on a network: it is possible for individuals to be infected before they have the opportunity to vaccinate, and this is more likely to occur when *β* is very large.

## 4. Conclusions

Here we analyzed a game theory-derived model of vaccinating behavior on a social contact network where a Susceptible-Exposed-Infectious-Recovered (SEIR) infection is transmitted along edges from individuals to their neighbors. Each day, individuals analyze the expected payoffs from being vaccinated and not being vaccinated and choose the choice that appears to maximize their expected life years at that time. Existing edges can break up and be replaced by new edges connecting the nodes to other randomly chosen nodes, which models changes in social interactions and mobility of individuals.

We identified several types of dynamics that arise from the nonlinear feedback mechanisms inherent in this and many other behavior-incidence models. For example, we observe that the final number of dead individuals does not increase linearly with the probability of death due to infection, *d*_inf⁡_. This is partly because a rising risk of death from infection induces more individuals to vaccinate and thus prevents more fatalities than would otherwise be the case. Additionally, transmission is reduced due to an increase in the number of nodes that die before infecting others, since death due to infection can reduce the duration of infectiousness in our model. Because the outcome (number of dead individuals) varies in a less than linear way with the input parameter (*d*_inf⁡_), we termed this *outcome inelasticity*.

A similar effect has been observed in a game theoretical model of vaccinating behavior during a pandemic, where it was observed that the epidemic peak occurred at roughly the same time for a broad range of values of the transmission rate, due to the response of the population to the unfolding outbreak [31]. Therefore, although we have explored these phenomena in the context of a specific model, the ubiquity of nonlinear feedbacks in these models may apply to vaccination behavior-incidence models more generally. This would tend to apply whenever the population has the latitude to respond in a way that mitigates a more transmissible or more severe infection, especially if the negative feedback is very strong.

Along similar lines, we observed that vaccine coverage may be higher with a vaccine that is less effective or more dangerous. This occurs because if the vaccine is sufficiently unattractive, not all neighbors of an index case will become vaccinated. As a result, the infection can break through imperfect vaccinee rings and percolate through the network, boosting final size and final vaccine coverage. We also observed that for some parameter regimes, the variability in outcomes can be very high: some stochastic realizations yielded a result of rapid containment of the infection through ring vaccination, while other stochastic realizations at the same parameter values yielded a situation of widespread vaccination and moderate final epidemic sizes. This variability has implications for predicting the effectiveness of control policies, and may reflect the existence of critical thresholds in the model.

We assumed a hypothetical, self-limiting, highly symptomatic close contact infection for which a vaccine exists. Real-world analogs for this are rare, but perhaps the closest analog is smallpox, which was eradicated through ring vaccination, was spread through close contact, and was highly symptomatic. Some future emerging zoonotic diseases may also fit this description, particularly if shortage of vaccine supplies due to recent emergence of the pathogen requires public health to prioritize vaccination for close contacts of index cases.

We made several simplifying assumptions that could influence model dynamics and hence our conclusions. For instance, we assumed that individuals do not react to what is occurring beyond their immediate neighbors. We also assumed the parameters do not change during the simulations or vary between individuals, however, in sensitivity analysis we analyzed scenarios with heterogeneous parameter values, and found little qualitative difference in our results. Potentially, individuals could seek preemptive vaccination, especially if they are aware of infection in the neighbors of their neighbors. An individual's views of the mortality rates due to the vaccine or the infection may change due to events within the rest of the population, for example, any negative effects caused by the vaccine may cause a vaccine scare. Furthermore, individuals may decrease their contacts (e.g., children may stay at home from school during an outbreak, or avoid a daycare) for fear of the infection, or protect themselves in other ways, such as wearing protective masks [14]. A further limitation of our model is that individuals in reality may not know that their neighbor is infectious, whereas we assumed that the start of infectiousness coincides with the appearance of symptoms and that all neighbors correctly deduce infection in an infected neighbor. We assumed no heterogeneity in the edge turnover rate *σ* or edge weights, although both edge turnover rate and edge weight will vary according to relationship type. Finally, we neglect clustering in the network, although realistic social contact networks can exhibit a high degree of clustering. These areas represent opportunities for further research.

In conclusion, we have found that behavior-incidence dynamics of an SEIR-type infection on a social contact network can exhibit effects that are relevant to disease control efforts: stochasticity near critical thresholds can make the effectiveness of ring vaccination difficult to predict, and nonlinear feedback mechanisms can lead to policy resistance or outcome inelasticity, neither of which would be predicted by a model that did not take strategic interactions or behavioral responses to disease dynamics into account. Policy resistance is a well-studied phenomenon in behavior-incidence models [13, 16, 18]. In comparison, outcome inelasticity and outcome variability have been less studied in the context of behavior-incidence models, but given their relevance to assessing disease control strategies perhaps they should be studied more extensively.

## Figures and Tables

**Table 1:** List of parameters, baseline values, and definitions.

Parameter	Value	Note
*α*	40 life years	Payoff for continued susceptibility
*β*	0.02/day	Probability of transmission along an edge
*ϵ*	0.95	Vaccine efficacy
*ζ*	Variable	Proportion of network edges changed per day
*ν*	10 nodes	Mean node degree
*d*_inf⁡_	0.3	Probability of death due to infection
*d*_vac_	0.001	Probability of death due to vaccination
*I*_0_	10 nodes	Initial number of infectious nodes
*L*	40 life years	Baseline payoff
*N*	5000 nodes	Population size
Ω_*γ*_	4 days	Variance of latent period
Ω_*σ*_	4 days	Variance of infectious period
1/*γ*	12 days	Mean duration of latent period
1/*σ*	19 days	Mean duration of infectious period

**Table 2:** Trends in the final number of efficaciously vaccinated, susceptible, and dead nodes for each regime.

Regime	Final vaccinated	Final susceptible	Final dead
Successful ring vaccination	Low	High	Very low
Widespread vaccination	Moderate	Moderate	Low to moderate
No vaccination	None	Low	High

## References

[1] Bonanni P (1999). Demographic impact of vaccination: a review. *Vaccine*.

[2] Jones KE, Patel NG, Levy MA (2008). Global trends in emerging infectious diseases. *Nature*.

[3] Hughes V (2006). News feature: a shot of fear. *Nature Medicine*.

[4] Levin SA (1994). A thousand and one epidemic models. *Frontiers in Mathematical Biology*.

[5] Brauer F (2008). *Compartmental Models for Epidemics*.

[6] Keeling MJ, Eames KTD (2005). Networks and epidemic models. *Journal of the Royal Society Interface*.

[7] Brito DL, Sheshinski E, Intriligator MD (1991). Externalities and compulsary vaccinations. *Journal of Public Economics*.

[8] Bauch CT, Galvani AP, Earn DJD (2003). Group interest versus self-interest in smallpox vaccination policy. *Proceedings of the National Academy of Sciences of the United States of America*.

[9] Bauch CT, Earn DJD (2004). Vaccination and the theory of games. *Proceedings of the National Academy of Sciences of the United States of America*.

[10] Chen FH (2006). A susceptible-infected epidemic model with voluntary vaccinations. *Journal of Mathematical Biology*.

[11] Klein E, Laxminarayan R, Smith DL, Gilligan CA (2007). Economic incentives and mathematical models of disease. *Environment and Development Economics*.

[12] Galvani AP, Reluga TC, Chapman GB (2007). Long-standing influenza vaccination policy is in accord with individual self-interest but not with the utilitarian optimum. *Proceedings of the National Academy of Sciences of the United States of America*.

[13] Funk S, Salathé M, Jansen VAA (2010). Modelling the influence of human behaviour on the spread of infectious diseases: a review. *Journal of the Royal Society Interface*.

[14] Reluga TC (2010). Game theory of social distancing in response to an epidemic. *PLoS Computational Biology*.

[15] d’Onofrio A, Manfredi P, Poletti P (2011). The impact of vaccine side effects on the natural history of immunization programmes: an imitation-game approach. *Journal of Theoretical Biology*.

[16] Reluga TC, Galvani AP (2011). A general approach for population games with application to vaccination. *Mathematical Biosciences*.

[17] Bauch CT, Bhattacharyya S (2012). Evolutionary game theory and social learning can determine how vaccine scares unfold. *PLOS Computational Biology*.

[18] Sterman JD (2006). Learning from evidence in a complex world. *American Journal of Public Health*.

[19] Fine PEM, Clarkson JA (1986). Individual versus public priorities in the determination of optimal vaccination policies. *American Journal of Epidemiology*.

[20] Brilliant L (1985). *The Management of Smallpox Eradication in India*.

[21] Fenner F, Henderson DA, Arita I, Jezek Z, Ladnyi ID (1988). *Small-Pox and Its Eradication*.

[22] Perisic A, Bauch CT (2009). A simulation analysis to characterize the dynamics of vaccinating behaviour on contact networks. *BMC Infectious Diseases*.

[23] Perisic A, Bauch CT (2009). Social contact networks and disease eradicability under voluntary vaccination. *PLoS Computational Biology*.

[24] Liljeros F, Edling CR, Nunes Amaral LA, Stanley HE, Åberg Y (2001). Social networks: the web of human sexual contacts. *Nature*.

[25] Wylie JL, Jolly A (2001). Patterns of chlamydia and gonorrhea infection in sexual networks in Manitoba, Canada. *Sexually Transmitted Diseases*.

[26] May RM, Lloyd AL (2001). Infection dynamics on scale-free networks. *Physical Review E*.

[27] Ferrari MJ, Bansal S, Meyers LA, Björnstad ON (2006). Network frailty and the geometry of herd immunity. *Proceedings of the Royal Society B*.

[28] Shaw LB, Schwartz IB (2008). Fluctuating epidemics on adaptive networks. *Physical Review E*.

[29] Funk S, Gilad E, Watkins C, Jansen VAA (2009). The spread of awareness and its impact on epidemic outbreaks. *Proceedings of the National Academy of Sciences of the United States of America*.

[30] Meyers LA, Pourbohloul B, Newman MEJ, Skowronski DM, Brunham RC (2005). Network theory and SARS: predicting outbreak diversity. *Journal of Theoretical Biology*.

[31] Bhattacharyya S, Bauch CT (2011). ‘Wait and see’ vaccinating behaviour during a pandemic: a game theoretic analysis. *Vaccine*.

